# Attitudes and experiences of nurses and carers with the carer support needs assessment tool intervention (CSNAT-I)

**DOI:** 10.1186/s12912-025-03960-7

**Published:** 2025-11-04

**Authors:** Camilla Anker-Hansen, Siv-Helene Østnes, Kine Torgersen, Elisabeth Bjørnstad Karlsen, Ann Karin Helgesen, Vigdis Abrahamsen Grøndahl

**Affiliations:** 1 Faculty of Health, Welfare and Organisation, Østfold University College, P.O. Box 700, Halden, N-1757 Norway; 2 Sykehuset Østfold Kalnes, Kalnesveien 300, Grålum, 1714 Norway; 3 Fredrikstad Kommune, Jens Wilhelms Gate 1, Kråkerøy, 1671 Norway; 4 UiT the Arctic University of Norway, Tromsø, Norway

**Keywords:** Caregiver needs, Carer support, CSNAT-I, Family caregivers, Nursing, Palliative care, Person-centred, Qualitative study

## Abstract

**Aim:**

This study explores the perspectives of nurses and informal carers regarding the implementation and use of the Carer Support Needs Assessment Tool Intervention (CSNAT-I) in palliative cancer care.

**Background:**

Carers play a crucial role in supporting patients with serious illnesses, yet their own support needs often remain unmet. The CSNAT-I was developed to systematically assess, address, and follow up on the needs of carers, aiming to enhance support for them.

**Method:**

Semi-structured interviews were conducted with three oncology nurses (via telephone with detailed field notes) and one focus group with four palliative-care nurses, alongside five carer interviews. Data were collected between January and March 2021, with all carer interviews and the focus group audio-recorded and transcribed verbatim. Data were analysed using content analysis, and the study adhered to COREQ reporting guidelines.

**Results:**

Although nurses acknowledged the importance of supporting carers, the integration of CSNAT-I was hindered by time constraints, unclear implementation processes, and limited understanding of its purpose. Both nurses and carers preferred ongoing dialogue and relational continuity over structured assessments. The CSNAT-I was often perceived as a conversation starter rather than a tool to guide support systematically. While some carers received practical help after the assessment conversation, they did not necessarily associate this support with the CSNAT-I.

**Conclusion:**

The study highlights key challenges with the CSNAT-I, such as issues related to timing, workload pressures, and limited awareness of its intended purpose. Carers emphasised the importance of continuous support and consistent contact points with healthcare professionals rather than relying solely on structured assessments. While CSNAT-I appears to play a valuable role in legitimising time spent with carers, its full potential is likely to be realised better if it is embedded within person-centred pathways that include clear strategies for implementation and follow-up.

**Supplementary Information:**

The online version contains supplementary material available at 10.1186/s12912-025-03960-7.

## Introduction

Being an informal carer for critically ill and dying individuals is often profoundly challenging [1]. Advancements in personalised treatments have extended the lives of those with terminal cancer, thereby prolonging the palliative care phase [2]. Carers, most often relatives, play an indispensable role in this journey. Beyond handling day-to-day tasks, they often anchor the patient’s identity, bringing moments of hope and consolation in challenging times [2]. Carers often hold invaluable insights into the patient’s condition, which becomes crucial when patients can no longer communicate effectively [3]. Such insights can play a pivotal role in enhancing and individualising care for the patient [4]. Moreover, carers often offer unwavering support throughout the treatment, both in institutional settings and at home. However, the ongoing responsibilities of caregiving can gradually impact the carer’s well-being [5]. One study identified over 200 challenges carers face, ranging from sleep disturbances and fatigue to pain [6]. Many also experience isolation due to diminishing social networks, leaving them to shoulder caregiving responsibilities alone [6]. Interactions with healthcare systems can exacerbate feelings of invisibility among carers, as they are often overlooked in clinical encounters where the focus remains exclusively on the patient, leaving carers’ own needs unacknowledged and unmet [7]. However, when involved collaboratively in the patient’s treatment by nursing staff, many carers report reduced anxiety and increased confidence in their role (4;6). Numerous studies repeatedly call for nurses to invest more time and expertise in carers, ensuring they feel acknowledged, heard, and included in the care planning for the care recipients ( [8, 9]). The studies however, lack a structured and standardised assessment tool for including the carer.

The World Health Organisation’s (WHO) definition of palliative care acknowledges the indispensable role of carers and the substantial challenges they encounter throughout the trajectory of the illness [10]. In Norway’s decentralised healthcare system, municipalities play a primary role in delivering care, including palliative care and home care services [11]. This distribution of responsibility can affect the continuity of care for carers, who may interact with both specialised and primary healthcare providers. In 2017, legislative changes in Norway formally recognised and emphasised the role of carers within health and care services [12]. That same year, the Norwegian Directorate of Health issued the ‘Relative Guidance,’ which concerns the involvement of and support for carers in health and care services [13]. The guide aims to ensure that carers receive the necessary support and guidance in practice. Among its recommendations, the government endorsed the CSNAT-I for a more systematic approach to addressing the support needs of carers during serious illnesses [13]. In 2021, the government introduced the “Carer Strategy” [14]. The strategy aims to recognise carers as a valuable resource and ensure comprehensive support so they can live fulfilling lives and balance their caregiving role with education and work. Furthermore, a person-centred approach to care has become a primary ambition in palliative care, aiming to enhance the quality of care for patients and their carers [15]. However, the same report highlights the inadequate support provided to carers. The follow-up measures are minimally structured, and the lack of support can negatively impact these carers’ health and employment participation [15].

The CSNAT-I is an evidence-based, person-centred assessment tool designed to identify and address the needs of carers supporting critically ill patients [16]. Its primary objective is to empower carers to articulate their unique needs and facilitate dialogue with healthcare professionals to develop tailored support measures. The tool encompasses 14 distinct domains where carers might require assistance, addressing both patient care and the carer’s well-being (17; see Table 1). According to Ewing et al. [18], the CSNAT-I consists of five stages (see Table 2).

**Table 1:** The CSNAT domains

○ Understanding your relative’s illness
○ Having time for yourself in the day
○ Getting a break from caring overnight
○ Managing your relative’s symptoms, including giving medicines
○ Your financial, legal or work issues
○ Providing personal care for your relative (e.g. dressing, washing, toileting)
○ Dealing with your feelings and worries
○ Knowing who to contact if you are concerned about your relative (for a range of reasons, including at night)
○ Looking after your own health (physical problems)
○ Equipment to help care for your relative
○ Your beliefs or spiritual concerns
○ Talking with your relative about his or her illness
○ Practical help in the home
○ Knowing what to expect in the future when caring for your relative

**Table 2:** The CSNAT-I approach [18]

Stage 1: Introduction	The CSNAT-I is introduced to the carer, preferably early in the caregiving process.
Stage 2: Carer’s consideration of needs	Carers are given time to consider their support needs.
Stage 3: Assessment conversation	Carers express and prioritise their support needs. Practitioners help identify needs and available support resources.
Stage 4: Shared action plan	The assessment leads to a shared action plan outlining actions for both the carer and practitioners to address identified support needs, including plans for future reviews.
Stage 5: Shared review	The review of the carer’s support needs is likely to be ongoing, with reassessments occurring during significant changes in circumstances, ensuring continuous support.

International research indicates that the CSNAT-I significantly reduces carer strain. Carers report feeling more acknowledged, having their needs better addressed, and perceiving enhanced overall support [19–22]. These international studies span different levels of evidence, encompassing robust quantitative research and consistent findings from qualitative studies, which together indicate positive effects for caregivers from the CSNAT intervention. Furthermore, the CSNAT-I effectively facilitates difficult conversations regarding end-of-life caregiving, especially during hospital discharge. In such situations, the organisational focus often remains on the patient, potentially overlooking the carer’s challenges [23].

While the CSNAT-I is used widely to facilitate structured, person-centred dialogues with carers, several other validated tools exist. For instance, a recent Taiwan-based study validated a Caregiver Strain Index-based model for quantifying caregiver burden in a long-term care context [24]. The 8-item Screening Tool for Family Caregiver Burden in Palliative Care (The CAREPAL-8) is a newly developed short screening tool designed to capture multiple dimensions of caregiver burden in palliative care [25]. Additionally, the Caregiver Self-Assessment Questionnaire (CSAQ) has demonstrated validity in detecting caregiver depression, underscoring its broader applicability as a self-screening measure [26]. Despite this toolkit, CSNAT-I remains unique in its emphasis on initiating a personalised needs assessment and subsequent dialogue in clinical practice.

In 2015, the CSNAT-I was translated into Norwegian and pilot-tested at Oslo University Hospital and St. Olav’s Hospital [27]. The implementation of the CSNAT-I in certain regions of Norway is detailed in an article by Hjermstad and Hanssen [28]. The Norwegian pilot study demonstrated that the translated CSNAT-I was well received by carers, who found the questions relevant, clear, and supportive of their role. Feedback indicated that the tool was perceived as particularly suitable for carers with daily or frequent caregiving responsibilities, and several emphasised the importance of using it as part of a dialogue rather than as a stand-alone form [27]. The subsequent implementation work in several palliative care centres in South-Eastern Norway reported positive experiences consistent with international findings [19–22], with CSNAT-I perceived as practical, useful, and well received by both carers and health care professionals. Most carers accepted the offer of a conversation and valued having their needs identified and followed up. These experiences have not yet been evaluated scientifically, and there remains a significant lack of empirical research on the utilisation and experiences of CSNAT-I within the Norwegian context [28], highlighting the need for studies that explore its implementation, impact, and adaptability.

### Purpose of the current study 

Given this context, this study aims to explore how nurses and carers perceive the implementation and practical use of CSNAT-I in palliative care. The study specifically focuses on facilitators, challenges, and their impact on carer support, addressing the following research question:

What are the perspectives of carers and nurses on the implementation and practical use of CSNAT-I in palliative cancer care?

### Methods

#### Design

The study employed a qualitative approach with an intensive design, meaning an in-depth exploration of a small number of strategically selected participants and settings to gain rich, detailed insights into their experiences and perspectives [29]. Data were collected through semi-structured individual interviews and a focus group interview.

#### Participants

The study was conducted in a hospital and associated municipal healthcare services in a mixed urban and semi-rural area in south-eastern Norway. Participants were recruited from an oncology ward and a mobile palliative care unit, with approval from the department head. Recruitment was facilitated by department managers, who identified nurses and carers with prior exposure to the CSNAT-I through either training or use in practice. In total, twelve participants were included: five carers and seven nurses. Participants were recruited using purposeful sampling to ensure a diverse range of perspectives relevant to the study aim.

#### Nurses

Three nurses from the oncology ward were invited to participate in individual interviews, and all agreed. Additionally, four nurses from the mobile palliative care unit were invited to a focus group interview and also agreed to participate.

The inclusion criterion was prior knowledge of the CSNAT-I through direct use or training. While oncology ward nurses had not used the CSNAT-I, they had received training in its application. The nurses from the mobile palliative care unit had completed training and had practical experience with CSNAT-I.

#### Carers

Five carers of patients receiving care from the mobile palliative care unit were invited for individual interviews, and all consented. The inclusion criterion was practical experience with the CSNAT-I.

To safeguard participants’ privacy and prevent identification within their local context, specific details, such as exact ages, are omitted. This measure was taken to protect participants’ identities and maintain confidentiality in reporting.

In the results section, nurses from the mobile palliative care unit (MPCU) are labelled as Nurses at MPCU (e.g., Nurse 1, MPCU), while nurses from the oncology ward (OW) are labeled as Nurses at OW (e.g., Nurse 1, OW). Carers are identified similarly (e.g., Carer 1).

### Data collection

The semi-structured interview guides were developed specifically for this study (available as a supplementary file), drawing on insights and frameworks from previous research [18] on CSNAT-I to ensure that the guides were grounded in existing knowledge of the topic. The guides provided a framework for the interviews while allowing flexibility to explore participants’ responses in depth as the conversation progressed. Follow-up questions were asked when necessary to gain a deeper understanding of the participants’ insights.

CAH conducted the focus group interview with nurses from the mobile palliative care unit at their workplace, which lasted one hour. The individual interviews with the carers were conducted face-to-face or via telephone, also by CAH, and each lasted between 30 min and one hour. One interview was conducted by phone due to COVID-19 restrictions. All interviews were recorded and subsequently transcribed for analysis.

The interviews with nurses at the oncology ward were conducted by SHØ. Due to the COVID-19 pandemic, these interviews were conducted over the phone. During each interview, notes were taken and subsequently transcribed after the session was completed. Each interview lasted approximately one hour.

All interviews were conducted between January and March 2021. Transcripts were not returned to participants for comment or correction, no interviews were repeated, and participants did not provide feedback on the study findings.

### Pre-understanding

The researchers involved in this study are all female and hold bachelor’s degrees in nursing. Additionally, all authors have personal and/or professional experience with caregiving, either as family members themselves or through providing services within healthcare settings. Three of the authors (CAH, AKH, VAG) have experience with qualitative research on the role and circumstances of carers in various contexts. This background provided them with a deep understanding of the challenges carers face.

The authors approached the research with the preconception that carers often encounter difficulties within the healthcare system and are frequently excluded from decisions affecting themselves and the individuals they care for. Based on this, they initially assumed that carers would perceive the CSNAT-I as a valuable tool for identifying their needs. However, during the analysis, they actively challenged this pre-understanding by seeking alternative interpretations and perspectives [29].

### Analysis

The data were analysed and synthesised by CAH and VAG through content analysis, following the methodology outlined by Graneheim and Lundman [30]. All interviews were read multiple times to gain a thorough understanding of the material. Text addressing the research question was extracted as the unit of analysis and divided into meaning units, which were identified based on connected words, sentences, or paragraphs within a consistent content and context. These meaning units were then condensed into shorter, more concise statements while preserving their alignment with the manifest content. Manifest content refers to the visible, surface meaning of the text, while latent content captures the underlying, implicit meaning [30].

Subsequently, the condensed meaning units were coded, and similar codes were grouped into three main categories and seven subcategories, maintaining alignment with the manifest content. In the final phase, these categories were analysed in relation to the latent content found in the interviews, which led to the development of an overarching theme [30]. CAH and VAG discussed the codes and final categories throughout the process, resolving minor disagreements through consensus. Once the analysis was complete, the remaining research group members were presented with the results. No further feedback was provided on the developed categories.

### Ethical considerations

Ethical approval was obtained from the Norwegian Centre for Research Data (NSD), reference number 430,636. Data collection adhered to the guidelines of the Helsinki Declaration [31]. Prior to the interviews, all participants received written and verbal information about the study’s purpose and the intended use of the results. The information also outlined the implications of participation and the participants’ right to withdraw at any time without penalty. Informed consent was obtained from all participants through the signing of a consent form. To ensure confidentiality, participants’ names were replaced with codes in all notes and transcriptions.

## Results

### Participant characteristics

Twelve participants took part in the study: five carers and seven nurses. The carers (four females, one male) had a median age of 67 years and were all spouses of patients with advanced cancer. The nurses (all female) were aged 20–51 years (median 49 years) and had between 2 and 21 years of work experience (median 13 years). All nurses worked in 80–100% positions.

### Overview of findings

The analysis revealed the latent theme: “The essential role of a person-centred dialogue”, and three interrelated categories: (1) Elements of time, (2) Limited awareness of CSNAT-I and its impact, and (3) Flexible support through personal interaction (Table 3). The results will be presented in the following according to the latent theme, categories, and subcategories.

**Table 3:** Results

Latent theme	The essential role of a person-centred dialogue		
**Category**	Elements of time	Limited awareness of CSNAT-I and its impact	Flexible support through personal interaction
**Sub-categories**	• Time pressure and balancing responsibilities • Timing and selection of implementation	• Limited process awareness • Unrecognised impact on carer support	• Person-centred support over structured forms • Direct access to professional support

### The essential role of a person-centred dialogue

The latent theme “the essential role of a person-centred dialogue” emphasises that individualised interaction is crucial for providing and receiving meaningful support. This theme captures the flexible approach desired by both carers and nurses. A key element in navigating these challenges is the necessity for meaningful dialogue between nurses and carers, which is rooted in recognising that every situation is unique. As one nurse explained:

It’s about having the time and the ability to sit down with carers; it shouldn’t be that I have just half an hour for you, expecting you to open up, so I can pat myself on the back and say, ‘There, I’ve done my part.’ (Nurse 1, OW).

A carer echoed this sentiment, noting,

It’s nice to express how you think and feel about things, but I’ve always sensed that when it comes to carers, we’re not important. (Carer 4).

These reflections illustrate how adaptable, relational dialogue, responsive to the specific needs and dynamics of each relationship, underpinned the findings in this study. As the latent theme represents a higher level of abstraction, the quotations recur from the manifest content. This reflects how the latent analysis builds on the manifest findings, capturing not only what was said but also the underlying meaning across carers’ and nurses’ accounts.

### Elements of time

Various aspects of time were highlighted. Participants differed in how they viewed the optimal timing for introducing the CSNAT-I. Some nurses, particularly in oncology, favoured earlier use to address needs proactively, whereas several in palliative care stressed that carers were often approached too late, or conversely, before they could articulate their needs. Carers themselves expressed varied preferences, shaped by readiness, the patient’s illness stage, and previous experiences with healthcare. The time available, or the lack thereof, in daily routines limited what nurses and carers could achieve and monitor. This is addressed under the subcategory *Time pressure and balancing responsibilities*. The subcategory *Timing and selection of implementation* explores the challenges of identifying the most appropriate time in the illness trajectory for introducing the CSNAT-I.

#### Time pressure and balancing responsibilities

All the nurses emphasised the importance of addressing the needs of carers, yet they quickly pointed out that time was a challenge. One nurse noted that she found it difficult to even engage in a conversation with carers, as she feared it would consume time she didn’t have. The limited time available led some nurses to question the feasibility of implementing the CSNAT-I in the ward. They stressed that new assessment tools needed to be perceived as both useful and time-saving, ensuring they would not further deplete the already scarce time resources. One nurse shared her initial thoughts upon receiving training in the use of the CSNAT-I:

First, it’s about time and resources. That was what I thought on the course day—where will we find the time? I believe that’s a general attitude. I know this sounds a bit negative, but I think there might be attitudes like, ‘are we supposed to do even more?’ Some days you have more time, and other days it’s non-stop. (Nurse 1, OW).

Further, there were concerns about initiating the CSNAT-I with carers, knowing that they would likely be interrupted during the process and might not have enough time to complete the entire procedure:

You feel that when you start this, there has to be time for it, so that the carers get the time they need, and that bells aren’t ringing, and you don’t have to run off. You need time to be able to fulfill what the carers need. (Nurse 3, OW).

One nurse feared that the assessment conversation could become just another task to check off as completed, without having the opportunity to dedicate sufficient time to do it properly. She explained it this way:

It’s about having the time and the ability to sit down with carers; it shouldn’t be that I have just half an hour for you, expecting you to open up, so I can pat myself on the back and say, ‘There, I’ve done my part.’ (Nurse 1, OW).

The nurses noted that each nurse was responsible for their own patients, with no one available to assist with their patients if they were spending time with carers. Furthermore, the shifts varied greatly, making it difficult to plan assessment conversations:

Time is a constraint. In a ward, many things can often happen, and the time and resources can make it impossible to carry out the conversations with carers as planned. This can lead to the conversation being superficial, not in-depth as the carers need, and we are unable to progress. (Nurse 3, OW).

Another nurse at the oncology ward pointed out that the ward had many patients with significant care needs, emphasising that the time spent with carers should not come at the expense of patient care.

The nurses at the mobile palliative care unit found that although the carers considered the CSNAT-I useful, they also encountered time constraints and questioned how much time they should dedicate to it:

I’ve had several positive conversations where they [carers] found it very useful… What I find challenging, though, is the time—it takes quite a long time. We often must limit it because they open up, and it’s difficult to set boundaries on how long it can continue. So, that’s perhaps what I feel… How much time should we spend on it? (Nurse 1, MPCU).

The carers also experienced the pressures of time within the healthcare system. One carer expressed a specific need during the assessment conversation, noting that she lacked sufficient information and requested to speak with a doctor. Although the doctor had promised to call, she was later informed by a nurse at the mobile palliative care unit that the doctor hadn’t had the time:

I really missed getting more information, and one of the doctors in the oncology department promised to call me… The nurse was supposed to arrange it, and she had, but he didn’t… so it didn’t happen. Then the nurse said, ‘I talked to him, and he didn’t have the time.’ And I said, well… then we’ll just forget about it… we won’t worry about it… we shouldn’t bother them… they have so much to do. (Carer 1).

Another carer described how overwhelming her responsibilities were, highlighting the constant pressure of time which she linked to why she didn’t remember many details about completing the CSNAT-I form, and that she hadn’t filled it out as thoroughly as she should have:

I just keep thinking about what I need to do, and I go to do one thing but almost forget what I was supposed to do. (…). You feel like you’re running from one thing to the next… as soon as I sit down, he calls me. (Carer 2).

#### Timing and selection of implementation

Another challenge with CSNAT-I that emerged from the interviews was determining the appropriate timing for introducing the tool to carers. One nurse at the oncology ward felt that CSNAT-I was too comprehensive and therefore could not be initiated in their department:

I think that the CSNAT-I is an excellent tool, but in a ward like this, the patients are often in the advanced stages of illness. Completing a 100% CSNAT-I in this ward, I don’t think is feasible. However, we can always follow up on something that has been started but not initiate it ourselves. (Nurse 2, OW).

A nurse in the mobile palliative care unit, however, pointed out that by the time patients began receiving their services, it was too late to initiate the CSNAT-I:

The CSNAT-I is supposed to be done, but then the patient is actually so ill, and things change so quickly, so the carers really don’t have… it’s just too late to bring it up, really. (Nurse 1, MPCU).

When asked if carers should have been introduced to CSNAT-I at an earlier stage, the nurse confirmed this. Conversely, another nurse (Nurse 2, MPCU) argued that if the CSNAT-I was introduced too early, the carers might not have an understanding of what assistance they needed, indicating that these needs typically emerged later in the disease trajectory of the person they cared for. Another nurse concluded that it is impossible to determine the perfect timing, emphasising that it must be assessed in each individual situation based on the needs of each carer:

When is the right time? Well, that likely varies from person to person. (Nurse 3, MPCU).

A carer expressed that the CSNAT-I should have been introduced earlier in her husband’s illness trajectory:

The form was introduced a bit later in the illness processes. What would have been helpful is if it had been available when I was insecure and didn’t act as I probably should have, like contacting the doctor earlier. If it [CSNAT-I] had been in place back then, that’s where I see there’s a gap… that initial phase feels like walking, how should I put it… across a swamp before finding solid ground again. (Carer 3).

Another carer shared that, based on the assessment conversation with the nurse, home nursing services were brought in at a time he initially felt was too early. However, he later acknowledged that it had been the right timing:

But as I said, when we had that conversation and the time afterward, home care services were introduced, and it turned out to be the right time, even though I didn’t fully understand at first that they should be here (…) But now, looking back, I realise it was very good that they got involved when they did… I got to know them, and I felt secure with them at an early stage. (Carer 5).

### Limited awareness of CSNAT-I and its impact

Across both carers and nurses, there was limited awareness of the full CSNAT-I process and its intended follow-up. Carers often did not connect the tool to the support they received, and nurses described uncertainty about how to integrate it as outlined in the literature. These gaps in knowledge and process contributed to inconsistent use of the tool and reduced its perceived impact. This is reflected in the subcategory *Limited process awareness*, which describes unclear understanding and limited adherence to the intended procedure, and in *Unrecognised impact on carer support*, where carers struggled to link the CSNAT-I to tangible outcomes.

#### Limited process awareness

Both carers and nurses had limited understanding of the CSNAT-I process, and the procedure was often adapted or only partially implemented. As a result, the tool’s intended structure and follow-up were not consistently applied. Carers frequently completed the form without prior explanation, and nurses described uncertainty about its correct use. Follow-up after the initial assessment conversation was not systematic; carers could instead contact the mobile palliative care unit when needed. One carer mentioned that she had never heard of the CSNAT-I before being asked to fill out the form and recalled very little of what she had written:

I don’t think I filled it all out. I don’t think I answered all the questions. No, I don’t think so… maybe I should have had a bit more information about it (…) I’ll go home and read more and see if I find it. Maybe I’ll recall more when I start reading about it. I don’t even remember what it is, what it stands for, what we agreed on. (Carer 2).

She did not feel that CSNAT-I was a tool designed to assist her as a carer; instead, it felt more like an informal conversation with the nurse at the mobile palliative care unit about her situation. Another carer echoed this sentiment, stating that she did not perceive CSNAT-I as a systematic tool, describing it instead as merely a conversation where she could express her needs:

Yes, I just talked… about my needs. I just talked, so I didn’t feel like I was following any form (Carer 3).

Another carer mentioned that she found it easy to fill out the form, but her positive experience shifted when it came time to review it with the nurse:

Well, in a way, I thought it was fine, but when I sat there going through the conversation, I thought… is that it? Is it just a conversation and nothing more? (Carer 4).

Although she expressed disappointment that the interaction didn’t go beyond a conversation, she also appreciated the opportunity to share her feelings, as she rarely felt her own experiences were acknowledged:

It’s nice to express how you think and feel about things, but I’ve always sensed that when it comes to carers, we’re not important. (Carer 4).

This was consistent with another carer’s experience, as she felt that everything revolved around her husband, who was battling cancer:

Yes, if anything comes up, I have her phone number [to the mobile palliative care unit], or we’ve been there and talked to her. But nothing was really about me… it was all about him. (Carer 2).

This carer also shared that she had not had any contact with the mobile palliative care unit after having the assessment conversation with the nurse. Although she was informed that she could reach out to the mobile palliative care unit if needed, no follow-up plan was established, and she was only provided with a phone number to call.

One carer had a slightly different experience than the others. He mentioned that he had spent time understanding the purpose of the CSNAT-I, and once he did, he found it much easier to grasp what was being asked in the CSNAT form:

I think the important thing was to understand the purpose of why you were being asked these questions, I mean, what it is intended for, what they aim to achieve. You want to do things right and make it easy right away, but it’s not always easy to understand what is being asked in the questions, and then you can end up giving answers that go off on a tangent. (Carer 5).

When asked about the action plan and whether it had been revised, he vaguely recalled that an action plan had been written.

Several of the nurses appeared to share this experience with the carers, both regarding the confusion CSNAT-I could cause for the carers and their uncertainty about what was expected of them after the initial assessment conversation. One nurse described it this way:

…what comes afterward, I mean after the assessment conversation, I don’t think that’s very clear because by then you might have talked about potential actions, and then what happens after that… it’s a bit vague, and I sometimes wonder about it myself: where do we go from here? And when? It’s a bit up in the air, I feel. (Nurse 1, MPCU).

None of the nurses had ever revised the action plan as described in the CSNAT-I process. One nurse initially explained:

I haven’t ever experienced making a second CSNAT-I conversation; I’ve never done that, just the first one, and then the agreement has been that we’ll be in touch, or we’ll set up new appointments if there’s a need for another conversation, but I’ve never done a second one, no. (Nurse 3, MPCU).

The nurses explained that the reason for this was that they had gotten to know the carers after the assessment conversation and were in ongoing dialogue with them anyway, making the further steps in CSNAT-I unnecessary.

#### Unrecognised impact on carer support

The interviews revealed that carers often did not connect the CSNAT-I with the support they and the patient received. For instance, one carer shared that neither she nor her circumstances aligned with a standardised approach. Specific needs identified on the form—like medication management and securing adaptive equipment—were met, but she did not attribute these to the CSNAT-I process. Instead, she credited a familiar nurse at the mobile palliative care unit who facilitated these services. Similarly, another carer emphasised the excellent support she received from healthcare providers but couldn’t link this back to the CSNAT-I:

So, it… no, it’s completely invaluable, all the help you get when he [her husband] is so seriously ill… that you can receive so much help… it’s just incredible. (Carer 3).

Another carer, when asked if CSNAT-I made it any easier to be a carer, responded unequivocally:

No, I can’t say that! (Carer 1).

However, during the interview, she noted certain support arrangements put in place following the assessment conversation about CSNAT-I, although she did not associate them directly with the tool.

A nurse shared that she also experienced that carers did not necessarily recognise their involvement in the CSNAT-I but rather saw it as having a conversation with the nurse, and they were satisfied with that. She had not encountered anyone who had linked it to CSNAT-I or expressed satisfaction with that tool:

We can have conversations, and they are satisfied, mostly expressing gratitude for having time to be heard and seen, and all of that. However, I haven’t yet experienced anyone saying that they are pleased with having used this [CSNAT-I]. (Nurse 2, MPCU).

Furthermore, there was a consensus among the nurses at the mobile palliative care unit that they also had similar conversations with carers without using the CSNAT-I.

### Flexible support through personal interaction

Both carers and nurses emphasised that meaningful support depended on flexibility and personal connection rather than rigid adherence to structured tools. The value of tailoring support to the unique needs of each carer was a consistent finding across groups. This is reflected in the subcategory *Person-centred support over structured forms*, highlighting the preference for relational dialogue, and in *Direct access to professional support*, where carers described the reassurance and responsiveness that came from having ongoing, personal contact with healthcare professionals.

#### Person-centred support over structured forms

Many carers preferred a person-centred approach that enabled open and flexible engagement with a familiar nurse, as the CSNAT-I was sometimes perceived as overly structured. One carer described feeling that he dominated the entire assessment conversation, steering it towards the questions he needed answers to. He emphasised that the nurses were very skilled, and he noted there was a risk of missing important aspects when he directed the conversation in this way:

(…) So, you could say the danger is that if you take over the show yourself and steer the conversation, you might miss out on a lot, so you have to be very careful with that. But at least I feel that I got very good answers… and when I came out of there, I actually felt quite empowered. (Carer 5).

This aligns with the experiences of several nurses at the mobile palliative care unit, who reported that carers preferred conversing with them rather than completing the CSNAT-I form and discussing it in a structured manner. One nurse described it as follows:

‘I’m not a fan of forms; I would rather have a conversation’ - I have encountered this sentiment several times recently, even though I try to promote the advantages of the forms. Many carers simply cannot manage the additional cognitive load and struggle to prioritise these tasks. (Nurse 4, MPCU).

Another nurse noted that while carers were willing to engage in conversation, encouraging them to complete the CSNAT-I form was more challenging.

#### Direct access to professional support

For the carers, direct contact with the hospital and familiar nurses was invaluable. All carers emphasised the importance of having a phone number to reach a nurse who knew them and their situation, allowing their needs to be addressed promptly. One carer highlighted how reassuring it was to know she could call a nurse at the mobile palliative care unit if needed or reach out to a cancer coordinator for assistance:

I could just call the nurse at the mobile palliative care unit if there was something, or I call the cancer coordinator if I have questions. (…). If something happens, I know I can call them, and they will help me immediately. And if something more serious happens, I know they will admit him [to the hospital]. (Carer 1).

Another carer reinforced this point, emphasising how having a direct contact number and a familiar voice provided much-needed peace of mind:

It has been incredibly reassuring to have that number and to be able to call her if I had any inquiries. It allowed me to relax, knowing I could just reach out and ask. (Carer 3).

She also noted that the mobile palliative care unit served as a crucial link to the doctors, a connection that could otherwise be difficult to establish independently.

The importance of having a designated contact person was further underscored by the nurses at the mobile palliative care unit. One nurse observed that when discussing potential revisions to the CSNAT form, some carers expressed a preference for maintaining informal communication rather than undergoing structured follow-up assessments:

I’ve experienced that they, in a way, have said that if I’ve asked, like, ‘should we review it again at a later time,’ some have simply said, ‘no, can’t we just stay in touch as we go?’ So, that’s really what it comes down to. (Nurse 2, MPCU).

Another nurse at the mobile palliative care unit suggested that after the initial assessment conversation, the structured CSNAT-I might become redundant:

After we’ve gone through the CSNAT form and gotten to know each other, I often feel that we have an ongoing dialogue anyway and that we might not need the form anymore. (Nurse 1, MPCU).

## Discussion

The findings indicate that the use of the CSNAT-I in this study was challenged by the unclear timing of its introduction, perceived time constraints, and a lack of understanding regarding its purpose. Participants often regarded the CSNAT-I as vague and secondary to the importance of ongoing contact and continuous dialogue. The ability to maintain communication and engage in open conversations was highlighted as more valuable than a structured tool such as the CSNAT-I. This discussion aims to interpret these results and situate them within the broader landscape of relevant research.

Uncertainties regarding the optimal timing for introducing the CSNAT-I emerged as a key issue. While nurses expressed differing views, most considered it too late once the patient had started mobile palliative care services or had been hospitalised. Conversely, one nurse from the mobile palliative care unit argued that introducing it too early would mean that carers lacked awareness of their own needs. However, a carer in the study, who initially perceived the introduction of the CSNAT-I as premature, later acknowledged in hindsight that the timing had been appropriate. These findings align with previous research indicating that the CSNAT-I is often perceived as either “too early,” when carers are not yet prepared to focus on their own needs, or “too late,” when patients have already been discharged or have passed away [17, 32]. As one nurse from the mobile palliative care unit remarked, “When is the right time? Well, that likely varies from person to person.” These findings suggest that there is no universally optimal timing for the CSNAT-I; rather its implementation must be tailored to each carer’s circumstances, in line with person-centred principles [33].

However, a highly flexible approach also entails risks. The concept of diffusion of responsibility, as described by Darley and Latané [34], suggests that when accountability is unclear, essential tasks are more likely to be neglected. In a fragmented healthcare system, where patients transition between hospitals, ambulatory teams, and home care services, the likelihood of the CSNAT-I not being introduced increases if no one within the care pathway is explicitly assigned this responsibility. Given the substantial evidence that carers often feel unseen and unheard within the healthcare system ( [8, 35, 36]), the systematic integration of the CSNAT-I into relevant patient pathways should be carefully balanced with the need for a flexible approach, assuming that the CSNAT-I is regarded as a tool to be embedded in practice.

Nurses in both the oncology ward and the mobile palliative care unit expressed concerns that implementing the CSNAT-I would require time they already lacked. However, they also acknowledged routinely engaging in similar, informal conversations with carers, where needs and concerns were continuously addressed. A comparable paradox was identified by Austin et al. [17], where nurses voiced apprehensions about the time demands of the CSNAT-I while simultaneously conducting comparable discussions without the tool. In our study, the primary challenge was allocating dedicated time for structured review and documentation. By contrast, Austin et al. [17] highlighted concerns that explicitly addressing the carer’s needs separately from those of the patient might introduce additional issues requiring resolution.

A key finding is the limited understanding of CSNAT-I among the carers. Few recognised that the form was part of a structured intervention designed to support them in their carer role. When asked about the action plan and its revision, most were unaware of its specific function, although all recalled having had conversations with a nurse. Similar findings were reported by Kreyer et al. [37], who noted that the CSNAT-I was often perceived as a conversation starter rather than a structured intervention. This suggests that the opportunity for dialogue may be more valuable than the structured completion of the intervention itself. However, both this study and Kreyer et al. [37] indicate that, despite its structured format, the CSNAT-I retains a degree of flexibility, enabling it to accommodate the diverse needs of carers.

Another notable finding was that nurses in this study were also uncertain about the CSNAT-I process. While they were familiar with the first three steps, none had fully understood the subsequent steps of the intervention. Instead, they relied on ongoing dialogue as part of their routine practice, reinforcing the notion that the CSNAT-I is primarily perceived as a conversation starter [37]. However, other studies suggest that the CSNAT-I can function as intended, albeit with varying positive outcomes for carers. For instance, Marston et al. [38] found that the CSNAT-I enabled carers to systematically identify their needs and access additional support during the transition from hospital to home. Similarly, Norinder et al. [22] reported that the CSNAT-I facilitated communication between carers and nurses, fostering involvement, a sense of security, and opportunities for reflection. Likewise, Kisch et al. [39] found that both carers and nurses perceived CSNAT-I as feasible and recognised its potential to provide valuable support. Although these studies were conducted in different settings, they indicate that the CSNAT-I can effectively support carers when integrated into practice with a clear process and follow-up structure. Interestingly, some carers in this study did acknowledge receiving practical help after the assessment conversation, but did not associate this assistance with the CSNAT-I. This suggests that the success of the CSNAT-I may depend less on formal recognition and more on ensuring that support is effectively delivered.

Two critical questions arise: Why did the nurses have such limited knowledge of the CSNAT-I, and why did all but one carer in this study perceive CSNAT-I as just a form to complete? Austin et al. [17] suggest that practitioners may be hesitant to integrate structured carer assessments because they already address carers’ needs within existing practice, albeit in a constrained manner due to time pressures, fear of causing distress, and the tendency to conflate patients’ and carers’ needs. Their study highlights how practitioners may exert control over what is discussed, who is involved, and when these conversations take place, ultimately limiting carers’ ability to fully articulate their needs [17]. However, our findings do not indicate that nurses sought to maintain control over the assessment process. Rather, the issue seemed to stem from a limited understanding of the intervention itself. Diffin [40] emphasises that successful CSNAT-I implementation requires facilitation processes and organisational support—without these, the tool risks becoming an administrative task rather than a meaningful support mechanism for carers. In this study, nurses appeared to rely on their usual ways of engaging with carers rather than integrating CSNAT-I as intended. While we cannot determine the exact reasons for this, it is reasonable to assume that a lack of training or clear implementation strategies may have contributed to the inconsistent use of the intervention. This, in turn, may also explain why the carers had such a limited understanding of CSNAT-I. If the nurses themselves did not fully grasp the intervention, they were likely unable to guide carers in how to use it effectively, reinforcing the perception that CSNAT-I was merely another form to complete rather than a tool designed to support them. Use of structured implementation frameworks, such as the Consolidated Framework for Implementation Research (CFIR) [41] or the Integrated Promoting Action on Research Implementation in Health Services (i-PARIHS) ( [42, 43]), could help address these challenges by identifying contextual barriers, supporting facilitation, and promoting sustained integration of CSNAT-I into routine practice.

An interesting finding was that the most important form of support for the carers was access to a hospital phone number they could call when seeking guidance or practical assistance, particularly during crises or periods of disease progression. Equally important was the reassurance that the person answering the call was familiar with their situation and the patient’s condition, reinforcing a sense of continuity and psychological safety. This underscores the fundamental value of direct, accessible, and personalised support in addressing carers’ needs, as also highlighted in international palliative care literature, where designated telephone lines are described as a vital “lifeline” for carers, ensuring timely advice, emotional reassurance, and avoidance of unnecessary hospital admissions [8, 44–46].

Although the carers did not always explicitly remember the CSNAT-I, they all recalled engaging in conversations with nurses about their roles as carers, suggesting that the tool may act as a catalyst for relationship-building. Nurses, however, expressed concerns that strict adherence to the CSNAT-I could limit flexibility and demand time they perceived as already scarce. Nonetheless, the CSNAT-I may lend legitimacy to prioritising conversations with carers. Research highlights that nurses often face challenges such as staffing shortages, high workloads, and time constraints [47]. As nursing has increasingly become task-oriented, administrative and technical duties have taken precedence over the relational aspects of care [48]. A recent study suggests that this shift has led nurses to regard emotional support and fostering security as “non-professional” tasks, with conversations sometimes seen as conflicting with professional expertise [49]. This task-driven environment may impede holistic, person-centred approaches, underscoring the need for frameworks like CSNAT-I to validate the relational aspects of nursing practice. By framing carer conversations as a structured task, CSNAT-I may provide a rationale for allocating time to these essential discussions, aligning with its original purpose as a facilitator of meaningful dialogue, rather than a rigid assessment tool [18].

### Strengths and weaknesses of the study

One of the key strengths of this study is its focus on both carers’ and nurses’ experiences with the CSNAT-I, providing a nuanced understanding of how the tool functions in practice. By employing qualitative methods, we captured in-depth perspectives on the relational and structural aspects of carer support, shedding light on the practical and contextual factors influencing the implementation of CSNAT-I. The study also contributes to the limited research on how structured carer assessments are perceived and utilised in Norwegian healthcare settings.

However, the study has some limitations. It was conducted within a specific healthcare setting, which may affect the transferability of the findings. The study sample size was small, but the participants were strategically selected based on their experiences, which gave rich data. Self-reported data from carers and nurses may be subject to recall bias or social desirability bias. Furthermore, the study did not include direct observations of CSNAT-I use, which could have provided additional insights into its practical application. We also acknowledge that the researchers’ perspectives may have influenced data interpretation. To enhance transparency, we have outlined our preconceptions and how they may have shaped the analysis. Lastly, while the study highlights key aspects of CSNAT-I implementation, it does not capture long-term effects. Future research should explore how CSNAT-I can be embedded in different healthcare contexts over time.

## Conclusion

This study highlights that carers do not necessarily require a structured intervention, but rather a systematic follow-up process that fosters ongoing relationships with healthcare professionals. Consistently, establishing familiarity with providers and the opportunity to reach out when needed were identified as the most critical aspects of carer support. While the CSNAT-I plays a role in facilitating these interactions, it should not be seen as a standalone solution.

The findings suggest that the effectiveness of the intervention is contingent upon its integration into care pathways in a manner that legitimises time for meaningful conversations. By framing carer discussions as a structured yet flexible process, the CSNAT-I may help ensure that these interactions are prioritised in healthcare settings that are increasingly task-oriented. However, without a clear implementation strategy and designated responsibility for follow-up, there is a risk that the CSNAT-I may not be fully utilised or perceived as valuable by both carers and nurses.

In light of these findings, future research should investigate how the CSNAT-I can be systematically embedded in different healthcare contexts and how structured assessments can be combined with relational, person-centred approaches to better meet carers’ needs. Larger and more diverse samples of carers and nurses are also needed to strengthen and expand the findings of this study.

## Supplementary Information

Below is the link to the electronic supplementary material.

Supplementary Material 1

Supplementary Material 2

Supplementary Material 3

Supplementary Material 4

## Data Availability

The datasets generated and analysed during the current study are not publicly available due to confidentiality agreements with the participants and ethical restrictions. Data are available from the corresponding author (C.A.H.) on reasonable request and with appropriate ethical approval.

## Declarations

### Use of artificial intelligence

This article has benefited from the proofreading and language refinement services provided by OpenAI’s ChatGPT Scholar (OpenAI, 2025).
