# Supplementary material for: Attitudes and experiences of nurses and carers with the carer support needs assessment tool intervention (CSNAT-I)
Source: BMC Nurs. 2025 Nov 4;24:1366. doi: 10.1186/s12912-025-03960-7 (PMC12584497; doi:10.1186/s12912-025-03960-7)
Supplement: Supplementary file 1 — Supplementary Material 1 [file 12912_2025_3960_MOESM1_ESM.docx]

**Focus Group Interview Guide – Nurses**

**Before Using CSNAT-I**
• How did you prepare to use CSNAT-I?
• Did you receive sufficient information and training on how to use CSNAT-I?
• What was most important to you during the preparation phase?

**Introducing CSNAT**
• What are your experiences with introducing CSNAT-I to family caregivers?
• Did you encounter any challenges?
• Did you feel that family caregivers found it easy to understand what the CSNAT-I process involves?

**Conducting the Assessment Conversation**
• What are your experiences with conducting the assessment conversation with family caregivers?
• Did you face any challenges?
• Was there anything that surprised you during the conversation?
• Can you describe your experience conducting the assessment conversations? How did it affect you?
• How did you perceive the caregivers’ reception of the assessment?

**Developing/Following Up the Action Plan**
• How did you experience the process of developing an action plan?
• To what extent were you able to meet the needs of the caregivers?
• Was the action plan modified over time?
• Can you describe how the assessment has been used in your department’s work?
• Have you experienced that CSNAT-I contributes to more person-centred collaboration with caregivers? If so, how?
• Would you recommend using CSNAT-I with other caregiver groups? If so, why?
• Have you documented the content and implementation of CSNAT-I? If so, how?
• How would you assess caregivers’ satisfaction with the use of CSNAT-I?
