# Supplementary material for: Attitudes and experiences of nurses and carers with the carer support needs assessment tool intervention (CSNAT-I)
Source: BMC Nurs. 2025 Nov 4;24:1366. doi: 10.1186/s12912-025-03960-7 (PMC12584497; doi:10.1186/s12912-025-03960-7)
Supplement: Supplementary file 2 — Supplementary Material 2 [file 12912_2025_3960_MOESM2_ESM.docx]

**Interview Guide – Individual Interviews with Family Caregivers**

**Introduction to CSNAT-I**
• Were you familiar with CSNAT-I before it was introduced to you in this project?
• Did you receive sufficient information about how to use CSNAT-I before completing the form and identifying your own needs?
• What was most important to you during the introduction to CSNAT-I?

**Conducting the Assessment Conversation**
• Can you describe your experience of the assessment conversation?
• Did you encounter any challenges?
• Was there anything that surprised you during the conversation?
• How did you experience participating in the assessment conversation? How did it affect you?
• Was there anything you wish had been done differently in the conversation? If so, what?

**Development and Follow-Up of the Action Plan**
• How did you experience developing the action plan?
• Did CSNAT-I help you feel seen and heard as a caregiver? If so, in what way? Can you give examples?
• Did the action plan change over time?
• To what extent did you feel that your identified needs were met?
• Did you find that the tool made it easier for you to be a caregiver?
• Did the use of CSNAT-I contribute to improved collaboration between you and healthcare personnel?
• Would you recommend CSNAT-I for use with other caregiver groups? If so, why?
• Is there anything you wish had been different in the CSNAT-I process (e.g., use of the tool/form/follow-up, etc.)?
• Overall, how satisfied are you with the use of CSNAT-I?
