# Supplementary material for: Attitudes and experiences of nurses and carers with the carer support needs assessment tool intervention (CSNAT-I)
Source: BMC Nurs. 2025 Nov 4;24:1366. doi: 10.1186/s12912-025-03960-7 (PMC12584497; doi:10.1186/s12912-025-03960-7)
Supplement: Supplementary file 3 — Supplementary Material 3 [file 12912_2025_3960_MOESM3_ESM.docx]

**Interview Guide: Individual Interviews with Nurses**

**Questions about CSNAT-I**

***For all nurses:***
• What do you know about CSNAT-I?
• Have you received information or training on CSNAT-I?
• Have you used CSNAT-I?

***If you have used CSNAT-I:***
• How did you experience using CSNAT-I?
• What strengths would you highlight in using CSNAT-I?
• What weaknesses would you point out in using CSNAT-I?
• Did you encounter any challenges? If so, why were these aspects challenging?
• How did you experience the time involved in using CSNAT-I?

***If you have not used CSNAT-I:***
• What are your thoughts on using it? Why?
• What is your general attitude toward adopting new assessment tools?
• What needs to be in place to make CSNAT-I easy to use?
• How do you feel about adopting or being introduced to a new tool?
• What barriers do you think may prevent the implementation of CSNAT-I in your unit?
• Do you think CSNAT-I could contribute to a more person-centred collaboration with family caregivers? If so, how?
• Do you think CSNAT-I could contribute to more systematic collaboration with family caregivers?
• What potential benefits do you see in using CSNAT-I?
• What limitations do you see in using CSNAT-I?
